# Chemical Constituents of Kino Extract from *Corymbia torelliana*

**DOI:** 10.3390/molecules191117862

**Published:** 2014-11-04

**Authors:** Motahareh Nobakht, Tanja Grkovic, Stephen J. Trueman, Helen M. Wallace, Mohammad Katouli, Ronald J. Quinn, Peter R. Brooks

**Affiliations:** 1 Genecology Research Centre, Faculty of Science, Health, Education and Engineering, University of the Sunshine Coast, Maroochydore DC, Queensland 4558, Australia; 2 Eskitis Institute for Drug Discovery, Griffith University, Nathan, Queensland 4111, Australia

**Keywords:** *Corymbia*, *Eucalyptus*, NMR spectroscopy, kino, natural products

## Abstract

Seven flavanones were identified from kino exudate of *Corymbia torelliana* by spectroscopic and spectrometric methods including UV, 1D and 2D NMR and UPLC-HR-MS. The study identified seven molecules, namely 3,4',5,7-tetrahydroxyflavanone (**1**), 3',4',5,7-tetrahydroxyflavanone (**2**), 4',5,7-trihydroxyflavanone (**3**), 3,4',5-trihydroxy-7-methoxyflavanone (**4**), (+)-(2*S*)-4',5,7-trihydroxy-6-methylflavanone (**5**), 4',5,7-trihydroxy-6,8-dimethylflavanone (**6**) and 4',5-dihydroxy-7-methoxyflavanone (**7**) from this eucalypt species. This is the first report of these natural products from *C. torelliana* kino exudate.

## 1. Introduction

*Corymbia torelliana* belongs to the Myrtaceae, a family of plants with at least 133 genera and more than 3800 species [1]. This family includes the eucalypts, *Eucalyptus*, *Corymbia* and *Angophora*, which are the world’s most widely planted hardwood trees. *C. torelliana* is native to rainforest fringes in tropical Australia and it has an unusual mutualism with stingless bees. Stingless bees are strongly attracted to the resin of *C. torelliana* fruits and the bees subsequently help to disperse the seeds [2,3,4,5], thereby making this tree species invasive where it has been introduced for amenity plantings outside its natural range.

Kino is a class of wood exudate found in many Myrtaceae species including the eucalypts. Kinos are characterized by their deep rich colouration, high tannin content, polyphenols and astringency [6,7]. Kinos have been used by Aboriginal people in Australia for thousands of years for medicinal purposes [8]. The secondary metabolite chemistry of kinos was initially examined 50 years ago by paper partition chromatography and two-dimensional co-chromatography and, more recently, by NMR, although only a small number of eucalypt species have been studied [9,10,11,12,13,14,15,16]. Different classes of phenolic compounds have been identified from kino extracts, and these compounds include aromadendrin, kaempferol, ellagic acid, gallocatechin, epicatechin, catechin, naringenin, hemiphloin, isohemiphloin, leucoanthocyanin, gallic acid and englitin [9,10,11,12,13,14,15,16]. In a recent study using NMR, 3,5,4',5''-tetrahydroxy-7-methoxy-6-[1-(p-hydroxy-phenyl)ethyl] flavanone and 3,5,7,4',5''-pentahydroxy-6-[1-(p-hydroxy-phenyl)ethyl] flavanone have been identified from kino exudate of *C. citriodora*—the first record of phenylethyl-dihydro flavonols in plant exudates [9].

We have found that crude extract from the kino of *C. torelliana* has antimicrobial activity against both Gram-negative and Gram-positive bacteria *in vitro*. This finding led us to isolate and characterise the major chemical components of *C. torelliana* kino extract with a view to future screening of their antimicrobial activities.

## 2. Results and Discussion

**Figure 1:**
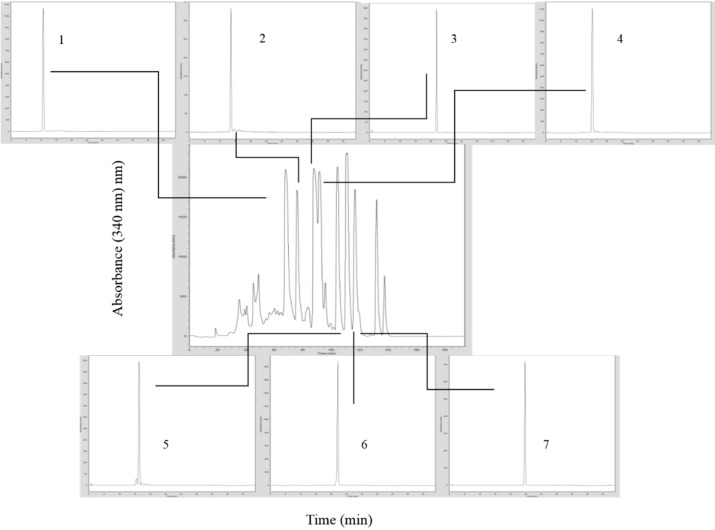
HPLC-UV/DAD chromatogram of crude extract from kino of *Corymbia torelliana*. UV trace of crude extract (with UV spectra of each major peak).

The crude extract of kino displayed a HPLC-UV trace of eight major compounds with flavonoid-like spectra (Figure 1). Seven compounds were recovered in sufficient purity and quantity by preparative HPLC for spectroscopic identification.

**Figure 2:**
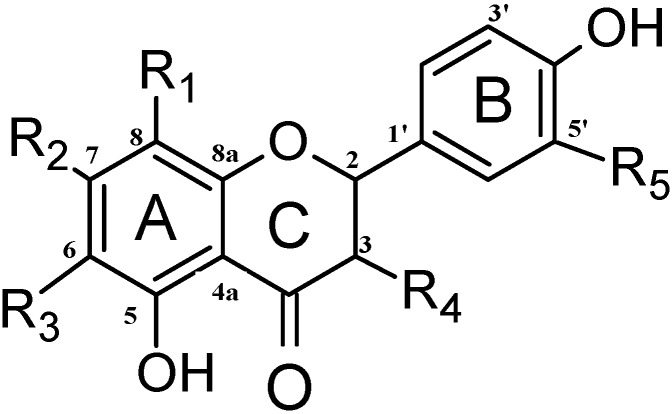
Structures of isolated flavanones (**1**–**7**) identified from kino of *Corymbia torelliana.*

The isolated compounds were identified via spectroscopic analyses, including ^1^H-NMR, ^13^C-NMR and high resolution mass spectroscopy. The NMR data and assignments for identified compounds are provided (Table 1, Table 2 and Table 3). The chemical formulas of the compounds were determined by liquid chromatography coupled to the high resolution mass spectrometry of [M–H]^−^ ions (Table 4). The structures of these compounds are shown (Figure 2).

All seven flavanones isolated from kino extracts of *C. torelliana* have a 4',5-dihydroxyflavanone core structure that is further substituted with hydroxyl, methoxy and/or methyl groups. Compounds were identified respectively as 3,4',5,7-tetrahydroxyflavanone (**1**), 3',4',5,7-tetrahydroxyflavanone (**2**), 4',5,7-trihydroxyflavanone (**3**), 3,4',5-trihydroxy-7-methoxyflavanone (**4**), (+)-(2*S*)-4',5,7-trihydroxy-6-methylflavanone (**5**), 4',5,7-trihydroxy-6,8-dimethylflavanone (**6**) and 4',5-dihydroxy-7-methoxyflavanone (**7**). All compounds were cream- to pale yellow-coloured powders, and all compounds are reported from the kino extract of this species for the first time.

3,4',5,7-tetrahydroxyflavanone (**1**) has been identified previously from kino exudates of *C. calophylla* and *C. gummifera* [13,14]. 4',5,7-trihydroxyflavanone (**3**) has been reported as naringenin from kino exudate of *C. maculata* [17], while 3,4',5-trihydroxy-7-methoxyflavanone (**4**) has been reported from kino exudates of *C. citriodora* [18]. Interestingly, only these three compounds have been reported previously from kino samples of eucalypt species. We are reporting four other known compounds for the first time from kino exudates, although these have been reported previously from other plant extracts. 3',4',5,7-Tetrahydroxyflavanone (**2**), *i.e*., eriodictyol, was isolated and identified for the first time from leaves of *Eriodictyon californicum* (Boraginaceae) [19]. (+)-(2*S*)-4',5,7-Trihydroxy-6-methylflavanone (**5**) has been found in *Pseudotsuga* spp. (Pinaceae) [20,21] and 4',5,7-trihydroxy-6,8-dimethylflavanone (**6**) has been reported from bulbs of *Pancratium maritimum* (Amaryllidaceae) [22] and stems of *Bauhinia glauca* (Leguminosae) [23]. 4',5-Dihydroxy-7-methoxyflavanone (**7**) has been obtained from resin of *Xanthorrhoea* spp. (Xanthorrhoeaceae) [24].

**Table:** 

Compound	R_1_	R_2_	R_3_	R_4_	R_5_
1	H	OH	H	OH	H
2	H	OH	H	H	OH
3	H	OH	H	H	H
4	H	OMe	H	OH	H
5	H	OH	Me	H	H
6	Me	OH	Me	H	H
7	H	OMe	H	H	H

**Table 1:** ^1^H and ^13^C-NMR spectral data for compounds **1** and **2** from *Corymbia torelliana.*

Position	Compound 1 (d_6_ DMSO)			Compound 2 (CD_3_OD)		
	^13^C	^1^H	HMBC	^13^C	^1^H	HMBC
2	82.7 (CH)	5.04 (d,11.4)	3,4,8a,1',2'	80.4 (CH)	5.27 (dd, 12.6, 3.0)	1',2',6'
3	71.3 (CH)	4.56 (d,11.4)	2,4,1'	44.1(CH_2_)	A 3.05 (dd,17.0, 12.6)	2,4,1'
					B 2.69 (dd, 17.0, 3.0)	4
4	197.2 (C)			197.2 (C)		
4a	100.1 (C)			103.3 (C)		
5	163.1 (C)			165.1 (C)		
5-OH		11.9 br s				
6	96.0 (CH)	5.89 (d, 1.8 Hz)	4a,5,7,8	97.6 (CH)	5.88 (d, 1.7)	4a,7
6-Me						
7	167.1 (C)			169.0 (C)		
7-Me						
8	95.0 (CH)	5.83 (d,1.8 Hz)	4a,6,7,8a	96.5 (CH)	5.86 (d, 1.7)	4a,7,8a
8a	162.4 (C)			164.6 (C)		
1'	127.5 (C)			132.0 (C)		
2'				114.7 (CH)	6.91 br s	2,1',3',4',6'
3'				116.3 (CH)	6.78 s	1'
4'	157.6 (C)			146.6 (C)		
5'				143.2 (C)		
6'				119.3 (CH)	6.78 s	2,2',4'
2'/6'	129.3 (CH)	7.31 (d, 8.25)	2,1',3'/5',4'			
3'/5'	114.8 (CH)	6.78 (d, 8.25)	1',2'/6',4'			

**Table 2:** ^1^H and ^13^C-NMR spectral data for compounds **3** and **4** from *Corymbia torelliana.*

Position	Compound 3 (d_6_ DMSO)			Compound 4 (CD_3_OD)		
	^13^C	^1^H	HMBC	^13^C	^1^H	HMBC
2	78.4 (CH)	5.43 (dd, 12.9, 2.9)	3,4,1',2'	83.7 (CH)	5.01 (d, 11.4)	3,4,1',2'
3	41.9 (CH_2_)	A 3.25 (dd, 17.2, 12.9)	2,4,1'	72.3 (CH)	4.57 (d, 11.4)	2,4,1'
		B 2.68 (dd, 17.2, 2.9)	2,1'			
4	196.8 (C)			197.6 (C)		
4a	101.7 (C)			101.2 (C)		
5	162.9 (C)			163.7 (C)		
5-OH		12.13 (br s)	4a,5,6			
6	95.8 (CH)	5.85 (br s)	5,7,8,8a	94.6 (CH)	6.08 (d. 2.4)	5,7,8,4a
6-Me						
7	166.7 (C)			168.4 (C)		
7-Me				54.9 (CH_3_)	3.8	7
8	94.9 (CH)	5.85 (br s)	5,7,8,8a	93.6 (CH)	6.04 (d, 2.4)	4a,6,7,8a
8a	163.4 (C)			163.0 (C)		
1'	128.8 (C)			127.7 (C)		
2'						
3'						
4'	157.6 (C)			157.8 (C)		
5'					6.8	
6'					7.35	
2'/6'	128.3 (CH)	7.31 (d, 8.5)	2,1',3'/5',4'	128.4 (CH)	7.35 (d, 8.4)	2,3',4'
3'/5'	115.1 (CH)	6.79 (d,8.5)	1',2'/6',4'	114.7 (CH)	6.80 (d, 8.4)	1',2',4'

**Table 3:** ^1^H and ^13^C-NMR spectral data for compounds **5**–**7** from *Corymbia torelliana.*

Position	Compound 5 (d_6_ DMSO)			Compound 6 (CD_3_OD)			Compound 7 (CDCl_3_)		
	^13^C	^1^H	HMBC	^13^C	^1^H	HMBC	^13^C	^1^H	HMBC
2	77.9 (CH)	5.32 (dd,12.4,2.8)	3,4,1',2'	78.6 (CH)	5.30 (dd, 12.9, 3.0)	1',2'	79.0 (CH)	5.34 (dd, 13.0, 3.0)	3,4,1',2'
3	42.0 (CH_2_)	A 3.12(dd,17.0,12.4) B 2.60 (dd,17.0,2.8)	2,4,1' 2,1'	42.7(CH_2_)	A 3.05 (dd,16.8, 12.9) B 2.71 (dd, 16.8, 3.0)	2,4,1' 4	43.2 ( CH_2_)	A 3.08 (dd,17.3, 13.0) B 2.78 (dd, 17.3, 3.0)	2,4,1' 2,1'
4	194.3 (C)			196.7 (C)			196.2 (C)		
4a	100.6 (C)			102.8 (C)			103.9 (C)		
5	160.4 (C)			157.9 (C)			164.5 (C)		
5-OH		12.47 (br s)	4a,5,6						
6	103.3 (C)			103.5 (C)			95.1 (CH)	6.06 (d, 2.4)	4a,5,7,8
6-Me	7.1 (CH_3_)	1.83 (s)	5,6,7	6.8 (CH_3_)	1.99 (s)	5,6,7			
7	168.8 (C)			163.6			168.1 (C)		
7-Me							55.7 (CH_3_)	3.79 (s)	7
8	94.9 (CH)	5.82 (s)	6,7,8a	101.6 (C)			94.2 (CH)	6.03 (d, 2.4)	6,7,8a
8-Me				6.0 (CH_3_)	1.98 (s)	7,8,8a			
8a	160.2 (C)			158.8 (C)			163.2 (C)		
1^'^	129.2 (C)			130.2 (C)			127.7(C)		
2'^'^/6'^'^	128.0 (CH)	7.29 (d, 8.3)	2,1',3'/5',4'	127.4 (CH)	7.32 (d, 8.4)	2,3',4'	127.9 (CH)	7.31 (d, 8.6)	2,1',3'/5',4'
3'^'^/5'	115.0 (CH)	6.77 (d, 8.3)	1',2'/6',4'	114.9 (CH)	6.81 (d, 8.4)	1',2',4'	115.7 (CH)	6.88 (d, 8.6)	1',2'/6',4'
4'	157.5 (C)			157.4 (C)			156.5 (C)		

**Table 4:** The kino extract of *Corymbia torelliana* contained flavanones **1**–**7**; analyses by UPLC-HR-MS.

Compound	Identification	Formula [M–H]^−^	Calculated Mass [M–H]^−^ (*m/z*)	Found Mass
1	3,4',5,7-tetrahydroxyflavanone	C_15_H_11_O_6_	287.05553	287.0565
2	3',4',5,7-tetrahydroxyflavanone	C_15_H_11_O_6_	287.05553	287.0566
3	4',5,7-trihydroxyflavanone	C_15_H_11_O_5_	271.06063	271.0616
4	3,4',5 -trihydroxy-7-methoxyflavanone	C_16_H_13_O_6_	301.07119	301.0721
5	(+)-(2*S*)-4',5,7-trihydroxy-6-methylflavanone	C_16_H_13_O_5_	285.07629	285.0773
6	4',5,7-trihydroxy-6,8-dimethylflavanone	C_17_H_15_O_5_	299.09695	299.0930
7	4',5-dihydroxy-7-methoxyflavanone	C_16_H_13_O_5_	285.07629	285.0771

## 3. Experimental Section

All solvents used for extraction and chromatographic analysis were analytical grade. Ethyl acetate (EtOAc), acetonitrile (ACN) and deuterated solvent (DMSO-*d*_6_) were purchased from Merck Pty Ltd (Kilsyth, Australia). Water was obtained from an in-house Milli-Q Ultrapure water system.

### 3.1. Plant Material

Fresh kino samples were collected from *C. torelliana* trees on the Sunshine Coast (26°42'S, 153°02'E), Queensland, Australia. The botanical identity of the trees was verified by Dr. Tom Lewis, Queensland Department of Agriculture, Fisheries and Forestry, and voucher specimens were deposited in the University of the Sunshine Coast herbarium (USC14055, USC14056 and USC14057). Samples were collected into clean vials from naturally occurring kino exudates of the trees from late November to March (late spring through early autumn), transported to the laboratory on ice, and stored in the dark at −20 °C until tested. Collection of samples from crystallized kino was avoided because of the effect of sunlight exposure on the chemical composition of kino [25].

### 3.2. Extraction

The kino samples were extracted in EtOAc/water (4:3). The EtOAc extracts were stored at −20 °C until fractionation by preparative HPLC. The EtOAc dry extract (100 mg) was dissolved in ACN/water (1:1) for fractionation by preparative chromatography. Eight compounds were recovered, but one compound was found in very low quantities and its identification has not been completed.

### 3.3. Chromatography

#### 3.3.1. Analytical HPLC-UV/DAD

Analytical chromatographic analyses were performed on a Perkin Elmer series 200 HPLC using Chromera software. The column was a Phenomenex Synergi Fusion 4 μm, 4.6 × 75 mm polar embedded RP column. Mobile Phase A was 95:5 water:ACN and Mobile Phase B was 10:90 water:ACN. The flow rate was 1.2 mL/min, gradient elution from mobile phase A to mobile phase B was 90/10 → 0/100, total run-time was 55 min, and detection was at 205, 260, 290 and 340 nm wavelength.

#### 3.3.2. Preparative HPLC

The EtOAc extracts were evaporated and re-dissolved (10% w/v) in ACN/water (1:1) and fractioned by preparative HPLC. Preparative chromatography was performed on an RP-C18 4 µm, 21.2 × 100 mm column (Fusion Phenomenex). Mobile Phase A was 95:5 water:ACN and Mobile Phase B was 10:90 water:ACN. The flow rate was 2 mL/min, gradient elution from Mobile Phase A to Mobile Phase B was 90/10 → 0/100, total run-time was 194 min, and detection was at 205, 260, 290 and 340 nm wavelength.

#### 3.3.3. UPLC-HR-MS Analysis

Samples from three *C. torelliana* kino samples were reconstituted in MeOH (2 mg/mL) and injected onto an Eclipse Plus C18 UPLC column 1.8 μm, 2.1 × 100 mm. Mobile Phase A consisted of 0.1% acetic acid in water and Mobile Phase B consisted of 0.1% acetic acid in ACN. The flow rate was 0.4 mL/min. The gradient was 2.5% Mobile Phase B at 0.5 min to 20% Mobile Phase B at 15 min and 100% Mobile Phase B at 25 min, then held for 2.5 min. A photodiode array detector was coupled to the LC and set at 205, 260, 290 and 340 nm. The spray voltage was set to 3.5 kV with the source temperature at 100 °C. The ESI module was Heated ESI (HESI) connected onto a qExactive Mass spectrometer (qEMS). The qEMS resolution was set at 140,000 and separate UPLC runs were performed for positive and negative ionization modes.

#### 3.3.4. Spectroscopic Analysis

NMR spectra were recorded on either a Varian 500 or 600 MHz unity INOVA spectrometer. The latter spectrometer was equipped with a triple resonance cold probe. The ^1^H and ^13^C-NMR chemical shifts were referenced to the solvent peaks for DMSO-*d*_6_ at δ_H_ 2.50 and δ_C_ 39.43, for CD_3_OD-*d*_4_ at δ_H_ 3.35 and δ_C_ 49.05 and for CDCl_3_-*d*_1_ at δ_H_ 7.25 and δ_C_ 77.0, respectively. Chemical shifts (δ) are given in ppm and coupling constants (*J*) in Hz (Table 1, Table 2 and Table 3).

## 4. Conclusions

We identified seven flavanones for the first time from kino exudate of the ecologically and ethnobotanically significant eucalypt tree, *Corymbia torelliana*. These compounds have a 4',5-dihydroxyflavanone core structure that is further substituted with hydroxyl, methoxy and/or methyl groups. Three of these flavanones have been reported previously from kino exudates of other *Corymbia* species, but we are reporting four other flavanones for the first time from eucalypt kino. The antimicrobial activity of these flavanones, and their potential role in the attractiveness of *C. torelliana* fruits to stingless bees, are the subjects of further study.

## References

[1] Wilson P.G., O’Brien M.M., Gadek P.A., Quinn C.J. (2001). Myrtaceae revisited: A reassessment of infrafamilial groups. Am. J. Bot..

[2] Leonhardt S.D., Wallace H.M., Schmitt T. (2011). The cuticular profiles of Australian stingless bees are shaped by resin of the eucalypt tree *Corymbia torelliana*. Austral Ecol..

[3] Wallace H.M., Howell M.G., Lee D.J. (2008). Standard yet unusual mechanisms of long-distance dispersal: Seed dispersal of *Corymbia torelliana* by bees. Divers. Distrib..

[4] Wallace H.M., Trueman S.J. (1995). Dispersal of *Eucalyptus torelliana* seeds by the resin-collecting stingless bee, *Trigona carbonaria*. Oecologia.

[5] Wallace H., Lee D. (2010). Resin-foraging by colonies of *Trigona sapiens* and *T. hockingsi* (Hymenoptera: Apidae, Meliponini) and consequent seed dispersal of *Corymbia torelliana* (Myrtaceae). Apidologie.

[6] Penfold A. (1961). The Eucalypts.

[7] Maiden J.H. (1901). The gums, resins and other vegetable exudations of Australia. J. R. Soc. N. S. W..

[8] Locher C., Currie L. (2010). Revisiting kinos—An Australian perspective. J. Ethnopharmacol..

[9] Freitas M.O., Lima M.A., Silveira E.R. (2007). NMR assignments of unusual flavonoids from the kino of *Eucalyptus citriodora*. Magn. Reson. Chem..

[10] Hillis W. (1963). The formation of polyphenols in trees 2. The polyphenols of *Eucalyptus sieberiana* kino. Biochem. J..

[11] Hillis W., Carle A. (1960). The formation of phenolic substances in *Eucalyptus gigantea* and *Eucalyptus sieberiana*. Biochemistry.

[12] Hillis W., Carle A. (1962). The chemistry of the eucalypt kinos iv. *Eucalyptus hemiphloia* kino. Aust. J. Chem..

[13] Hillis W. (1951). The chemistry of the eucalypt kinos. Part i. Chromatographic resolution. Aust. J. Basic Appl. Sci..

[14] Hillis W. (1952). The chemistry of the eucalypt kinos. Part ii. Aromadendrin, kaempferol and ellagic acid. Aust. J. Sci. Res. Ser. A.

[15] Hillis W., Carle A. (1960). The chemistry of the eucalypt kinos iii. (+)-Afzelechin, pyrogallol, and (+)-catechin from *Eucalyptus calophylla* kino. Aust. J. Chem..

[16] Lambert J.B., Wu Y., Kozminski M.A. (2007). Characterization of Eucalyptus and chemically related exudates by nuclear magnetic resonance spectroscopy. Aust. J. Chem..

[17] Gell R., Pinhey J., Ritchie E. (1958). The constituents of the kino of *Eucalyptus maculata* Hook. Aust. J. Chem..

[18] Satwalekar S.S., Gupta T.R., Narasimha Rao P.L. (1956). Chemical and antibacterial properties of kinos from *Eucalyptus* spp. Citriodorol—The antibiotic principle from the kino of *Eucalyptus citriodora*. J. Indian Inst. Sci..

[19] Power F.B., Tutin F. (1906). Chemical examination of Eriodictyon. Pharm. Rev..

[20] Barton G.M. (1967). A new c-methyl flavanone from diseased (*Poria weirii* Murr.) Douglas fir (*Pseudotsuga menziesii* (Mirb.) Franco) roots. Can. J. Chem..

[21] Yi J.H., Zhang G.L., Li B.G. (2002). Studies on the chemical constituents of *Pseudotsuga sinensis*. Acta Pharm.Sin..

[22] Youssef D.T.A., Ramadan M.A., Khalifa A.A. (1998). Acetophenones, a chalcone, a chromone and flavonoids from *Pancratium maritimum*. Phytochemistry.

[23] Wu Z.B., Zhao Y.Y., Yang X.Y., Liang H. (2009). Flavonoids from *Bauhinia glauca* subsp. *pernervosa*. Chem. Pharm. Bull..

[24] Birch A.J., Dahl C.J. (1974). Some constituents of the resins of *Xanthorrhoea preissii, australis* and *hastile*. Aust. J. Chem..

[25] Maiden J.H. (1889). Botany bay of eucalyptus kino. Pharm. J. Trans..

